# The choice of a postpyloric tube and the patient’s position in our procedure: A response

**DOI:** 10.1186/s13054-018-2036-7

**Published:** 2018-05-10

**Authors:** Bei Hu, Bo Lv, Chunbo Chen

**Affiliations:** Department of Critical Care Medicine, Guangdong General Hospital, Guangdong Academy of Medical Sciences, 106 Zhongshan Er Road, Guangzhou, 510080 Guangdong Province People’s Republic of China

In a recent letter published in *Critical Care* [1], Sun and colleagues argued that the choice of a postpyloric tube and the patient’s position with regard to spiral nasojejunal feeding tube insertion in a previously published study [2] required further improvement.

**Fig. 1:**
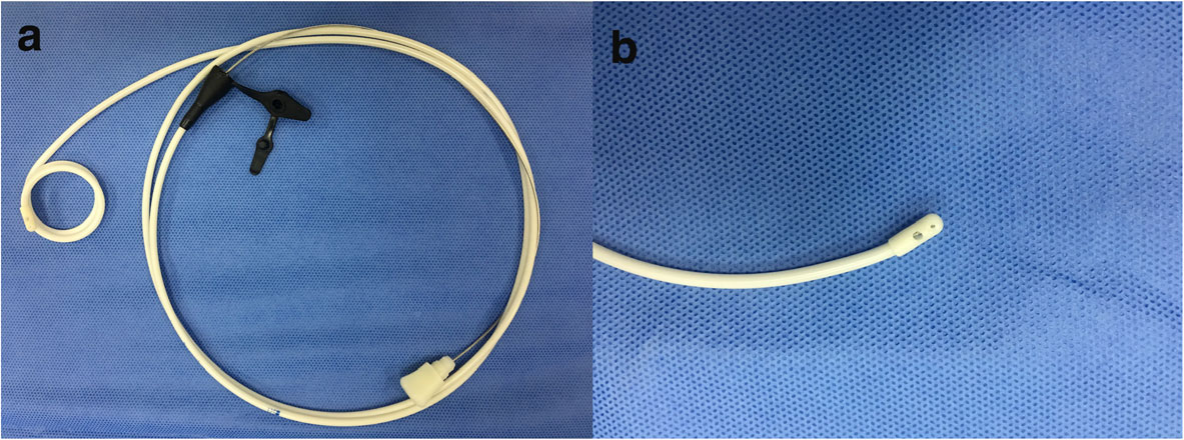
The 145-cm-long spiral feeding tube made of radiopaque polyurethane with a spiraled extremity (2.5 loops with a diameter of 3 cm, CH10, inner diameter 1.95–2.10 mm, Flocare Bengmark, Nutricia, The Netherlands) used in our center (**a**). This Flocare Bengmark tube has four side holes near its tip, and the blind end of the tube is blunt with a guide wire (**b**)

Both spiral and straight feeding tubes were used for blind bedside transpyloric tube placement [3]. In our study, a 145-cm-long spiral tube made of radiopaque polyurethane (CH10, Flocare Bengmark, Nutricia, The Netherlands) was used for rescue therapy subsequent to failed spontaneous transpyloric migration despite using prokinetic agents. This tube has a preformed spiral in the distal 23 cm (2.5 loops with a diameter of 3 cm; Fig. 1a), designed to utilize peristalsis for transpyloric migration. The specially spiral feature is promoted as an aid to spontaneous passage through the pylorus and maintenance in the duodenum or jejunum; straight tubes, however, might migrate back to the stomach due to duodenum anti-peristalsis movements. The procedure of spiral nasojejunal tube intubation is simple, rapid, well tolerated, and highly successful with little training. Moreover, complications are rare. Rapid tube reinsertion after failed migration is also feasible. The use of this tube is preferred in critically ill patients in our center due to the aforementioned advantages. In our experience, this technique has obviated the need to search for another method to insert a transpyloric feeding tube. It is appropriate to make full use of the same tube instead of using a new one, which may incur additional cost. In our procedure, it is an alternative procedure after failed spontaneous postpyloric migration rather than a first choice. The spiral tube that we used has four side holes near its tip (Fig. 1b). Therefore, it is not likely to be blocked under standard maintenance. Although the guide wire of the spiral tube is a little longer than that of the Flocare tube used by the authors, the flexibility was better in our experience. The blind end of the tube is blunt, and damage to the digestive tract during insertion is rare too.

When it comes to the patient’s position during the postpyloric placement of the nasojejunal tube, we consider the author’s procedure is quite creative. We are willing to make a comparison between the two positions in future work and optimize the method of remedial spiral feeding tube insertion. One position is described in the authors’ work [1], another position is described in our previous published work [2].

Our procedure of using the spiral nasojejunal tube as a preferred enternal nutrition method in critically ill patients is as follows: radioscopy confirmation is taken 24 h after successful gastric placement with or without use of prokinetic agents [4]. Blind bedside postpyloric placement of the spiral tube as rescue therapy is implemented after a failed transpyloric migration [2]. The expected success rate is more than 90% using this strategy. This cost-effective protocol can be readily and rapidly learned through an appropriate professional training course, regardless of previous experience. Further, a real-world study (ChiCTR-INR-16009099) [5] is planned to verify the procedure of postpyloric placement of the spiral nasojejunal feeding tube in critically ill adults.
